# The Efficacy of TACE Combined With Lenvatinib Plus Sintilimab in Unresectable Hepatocellular Carcinoma: A Multicenter Retrospective Study

**DOI:** 10.3389/fonc.2021.783480

**Published:** 2021-12-20

**Authors:** Fei Cao, Yi Yang, Tongguo Si, Jun Luo, Hui Zeng, Zhewei Zhang, Duiping Feng, Yi Chen, Jiaping Zheng

**Affiliations:** ^1^ Department of Interventional Radiology, The Cancer Hospital of the University of Chinese Academy of Sciences (Zhejiang Cancer Hospital), Institute of Basic Medicine and Cancer (IBMC), Chinese Academy of Sciences, Hangzhou, China; ^2^ Department of Hepatobiliary Surgery, National Cancer Center/National Clinical Research Center for Cancer/Cancer Hospital, Chinese Academy of Medical Sciences and Peking Union Medical College, Beijing, China; ^3^ Key Laboratory of Gene Editing Screening and Research and Development (R&D) of Digestive System Tumor Drugs, Chinese Academy of Medical Sciences and Peking Union Medical College, Beijing, China; ^4^ Department of Interventional Therapy, Tianjin Medical University Cancer Institute and Hospital, National Clinical Research Center for Cancer, Key Laboratory of Cancer Prevention and Therapy, Tianjin’s Clinical Research Center for Cancer, Tianjin, China; ^5^ Department of Interventional Radiology, First Hospital of Shanxi Medical University, Taiyuan, China

**Keywords:** hepatocellular carcinoma, transarterial chemoembolization, targeted therapy, immunotherapy, comprehensive therapy

## Abstract

**Objective:**

To assess the efficacy and safety of transarterial Chemoembolization (TACE) combined with lenvatinib plus sintilimab in unresectable hepatocellular carcinoma (HCC).

**Patients and Methods:**

The data of patients with unresectable HCC administered a combination therapy with TACE and lenvatinib plus sintilimab were retrospectively assessed. Patients received lenvatinib orally once daily 2 weeks before TACE, followed by sintilimab administration at 200 mg intravenously on day 1 of a 21-day therapeutic cycle after TACE. The primary endpoints were objective response rate (ORR) and duration of response (DOR) by the modified RECIST criteria.

**Results:**

Median duration of follow-up was 12.5 months (95%CI 9.1 to 14.8 months). ORR was 46.7% (28/60). Median DOR in confirmed responders was 10.0 months (95%CI 9.0-11.0 months). Median progression-free survival (PFS) was 13.3 months (95%CI 11.9-14.7 months). Median overall survival (OS) was 23.6 months (95%CI 22.2-25.0 months).

**Conclusions:**

TACE combined with lenvatinib plus sintilimab is a promising therapeutic regimen in unresectable hepatocellular carcinoma.

## Introduction

Hepatocellular carcinoma (HCC) is one of the most prevalent malignant tumors and the fourth leading cause of cancer-related death worldwide (1). In patients with early-stage HCC, ablation, resection and transplantation have been recommended as curable therapies (2). Although unresectable disease may be treated by transarterial chemoembolization (TACE) and systemic therapy, most patients have a poor prognosis (3).

According to the Barcelona Clinic Liver Cancer (BCLC) staging and treatment strategy, TACE represents the standard treatment option for patients with intermediate stage HCC (4), being associated with longer survival compared with best supportive care (5). Meanwhile, only TACE hardly improves survival in advanced HCC (6, 7).

Lenvatinib has become a first−line systemic therapeutic option for advanced HCC (3, 4). In the phase III REFLECT trial (8), lenvatinib was non-inferior to sorafenib in overall survival (OS), showed greater objective response rate (ORR) and median progression-free survival (PFS), and conferred hepatic function in advanced HCC (9, 10).

Immune checkpoint inhibitors, as immunotherapeutic agents, have shown promising outcomes in patients with advanced HCC (11). In the KEYNOTE-240 study, although OS and PFS did not reach statistical significance, ORR and overall response were better than those of placebo, and some patients benefited from pembrolizumab (12). Sintilimab is a human immunoglobulin G4 (IgG4) monoclonal antibody that specifically binds to the PD-1 molecule on the surface of T cells, consequently blocking the tumor immune tolerance-inducing PD-1/programmed death-ligand 1 (PD-L1) pathway, re-activating the anti-tumor activities of lymphocytes, and inhibiting tumors. Sintilimab has been approved for marketing in December 2018, mainly for the treatment of recurrent or refractory classical Hodgkin lymphoma previously treated with at least two lines of chemotherapy. Sintilimab is also been applied for the treatment of various solid tumors in clinical practice, including lung cancer, liver cancer, and esophageal cancer, with notable safety and high efficacy.

Combination therapies have been researched for liver cancer, with favorable results (13), including PD-1 inhibitors plus lenvatinib, TACE plus Sorafenib and TACE plus Lenvatinib (14, 15). However, to date, TACE combined with lenvatinib plus sintilimab has not been studied for patients with unresectable HCC. Therefore, we conducted this retrospective study to assess the efficacy and safety of TACE combined with lenvatinib plus sintilimab in unresectable HCC.

## Patients and Methods

### Patients

In this retrospective study, the medical records and imaging data of HCC patients were obtained at the Cancer Hospital of the University of Chinese Academy of Sciences, Tianjin Medical University Cancer Institute and Hospital, and First Hospital of Shanxi Medical University between January 2019 and March 2021. All patients were diagnosed with HCC by non-invasive criteria or biopsy. The non-invasive diagnostic criteria for HCC in patients with cirrhosis were: liver cirrhosis; tumor diameter larger than 1 cm based on four-phase multi-detector computed tomography (MDCT) or dynamic magnetic resonance imaging (MRI), and arterial hypervascularization with venous or delayed phase washout (16, 17). Inclusion criteria were: [1] BCLC B or C stage; [2] at least one measurable target lesion; [3] Eastern Cooperative Oncology Group Performance Status (ECOG-PS) score of 0-1; [4] Child-Pugh class A score of 5-6. Exclusion criteria were: [1] prior systemic therapy or immunotherapy; [2] follow up <6 months; [3] a history of autoimmune disease. This study was approved by the Ethics Committees of the Cancer Hospital of the University of Chinese Academy of Sciences, Tianjin Medical University Cancer Institute and Hospital, and First Hospital of Shanxi Medical University. All patients were required to provide written informed consent before inclusion in the study.

### TACE

TACE was performed by interventional radiologists (F.C, J.Z, T.S, Y.C) with more than ten years of experience. After puncturing the femoral artery, celiac trunk and superior mesenteric artery angiography were performed selectively with a 5F catheter (RH catheter; Cook, Bloomington, Ind). When the tumor-feeding arteries were found, the catheter was advanced into them one by one; a 3F microcatheter (SP microcatheter; Terumo, Tokyo, Japan) was used for selective catheterization if necessary. Oxaliplatin (75 mg/m^2^) was infused *via* the catheter, and iodized oil (Lipiodol Ultrafluido; Guerbet, Aulnay-sous-Bois, France) mixed with epirubicin (30-50 mg/m^2^) was used to embolize tumor-feeding arteries. The TACE procedure was repeated 4-6 weeks later.

### Systemic Therapy

Patients received lenvatinib at 12 mg (bodyweight >60 kg) or 8 mg (bodyweight < 60 kg) orally once daily 2 weeks before TACE (6). Patients were administered sintilimab at 200 mg intravenously on day 1 of a 21-day therapy cycle after the TACE procedure.

### Follow-Up Visits

Follow-up visits were performed 4-6 weeks after the TACE procedure. The patients underwent chest CT, abdomen multiphase CT or MRI, and laboratory examinations during each follow-up visit. The laboratory examinations encompassed liver function tests, including bilirubin, aspartate aminotransferase (AST), alanine aminotransferase (ALT), albumin (ALB) and γ-glutamyl transpeptidase (γ-GT) level assessment, along with prothrombin time (PT) and serum α-fetoprotein (AFP) level evaluation. OS was defined as the time from the first TACE treatment to death or the last follow-up. PFS was defined as the time from the first TACE treatment to disease recurrence or the last follow-up. Intrahepatic tumor progression (25% increase from baseline) and transient deterioration of liver function to Child-Pugh C, macrovascular invasion (MVI) or extrahepatic metastasis was considered to indicate disease progression (6).

### Statistical Analysis

Primary endpoints were objective response rate (ORR) and duration of response (DOR) determined by the modified RECIST criteria. The Kaplan-Meier method was used to estimate DOR, PFS and OS. Patients with confirmed complete response (CR) or partial response (PR) were analyzed for DOR. All statistical analyses were performed with SPSS version 23.0.

## Results

### Patient Demographics

Totally 60 patients were enrolled in the current study between January 2019 and March 2021. Six individuals were excluded because of prior sorafenib or lenvatinib or PD-1 treatment, and two were excluded for follow up <6 months; finally, 52 patients were analyzed (40 patients from Cancer Hospital of the University of Chinese Academy of Sciences, 6 patients from Tianjin Medical University Cancer Institute and Hospital, and 6 patients from First Hospital of Shanxi Medical University). Age, gender, Child-Pugh class, hepatitis B virus (HBV) infection ratio, alpha fetoprotein (AFP) levels, albumin-bilirubin (ALBI) score, BCLC stage and ECOG-PS score were examined. The baseline characteristics of the 60 patients were collected before therapy (**Table 1**).

**Table 1:** Baseline characteristics of the 52 patients.

Characteristic	NO.(%)
**Age (years)**	
≤65	40 (76.9%)
>65	12 (23.1%)
**Gender**	
Female	7 (13.6%)
Male	45 (86.4%)
**HBV infection**	
Yes	47 (90.4%)
No	5 (9.6%)
**Child-Pugh score**	
A	46 (88.5%)
B	6 (11.5%)
**AFP (ng/mL)**	
≤400	34 (65.4%)
>400	18 (34.6%)
**ALBI score**	
1	10 (19.2%)
2	40 (77.0%)
3	2 (3.8%)
**ECOG- PS**	
0	7 (13.5%)
1	45 (86.5%)
**BCLC stage**	
B	13 (25.0%)
C	39 (75.0%)
**Macroscopic vascular invasion**	19 (36.5%)
**Extrahepatic site**	21 (40.4%)
lung	10 (19.2%)
nodes	10 (19.2%)
bone	5 (9.6%)
other	3 (5.8%)

### Efficacy

**Figure 1:**
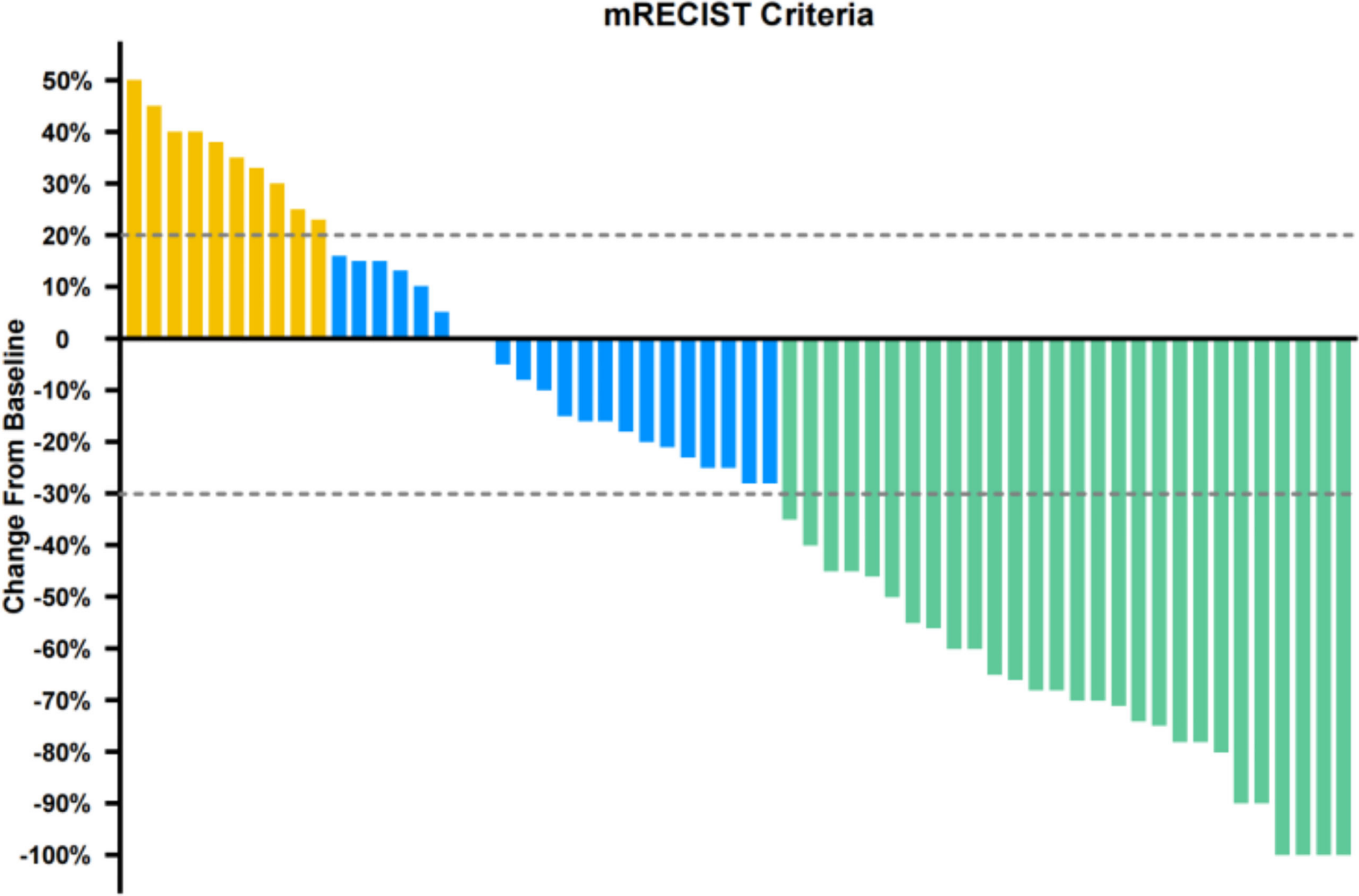
Percentage changes from baseline (summed diameters of target tumors by mRECIST).

**Figure 2:**
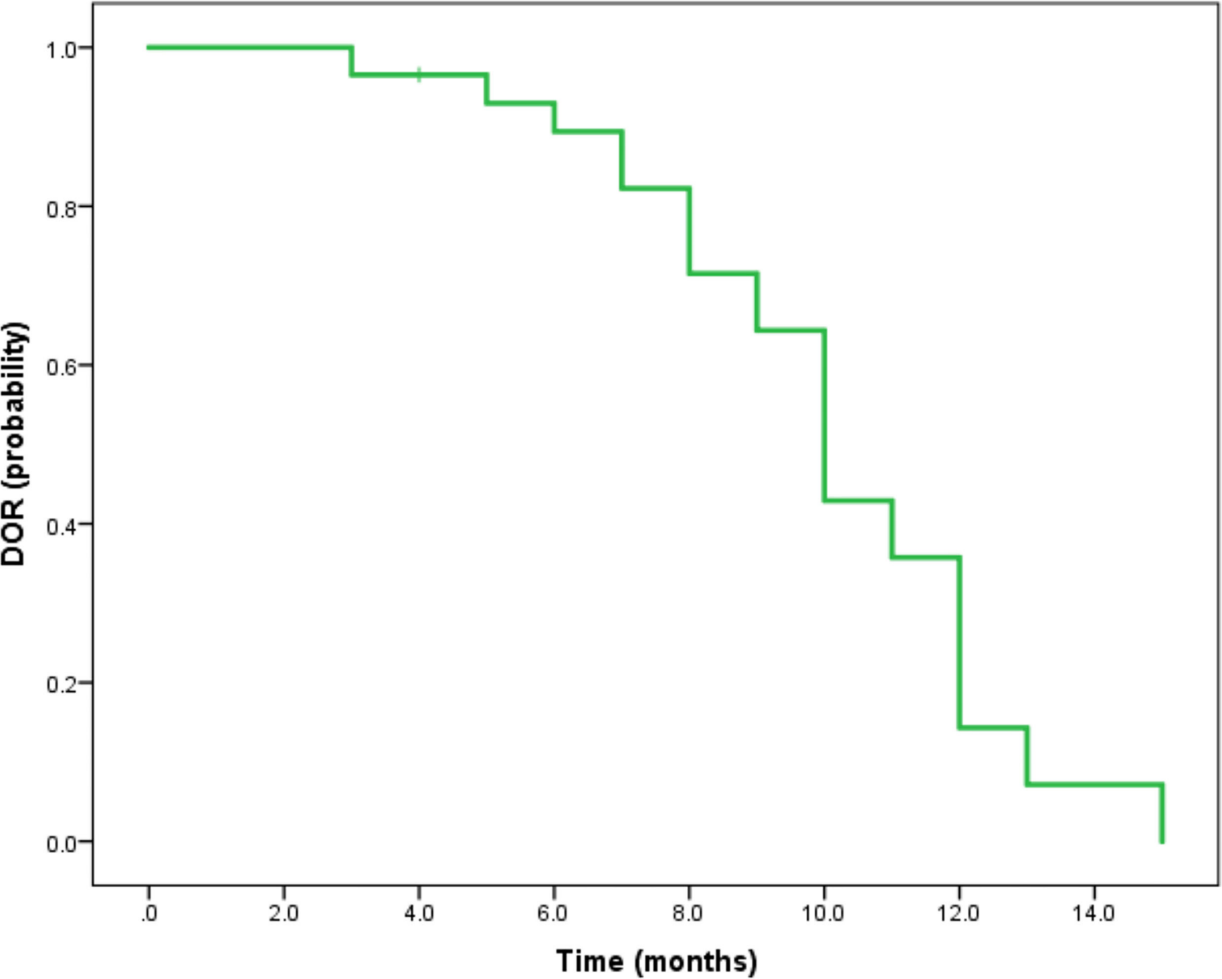
Kaplan-Meier method-based estimate of DOR by mRECIST.

**Figure 3:**
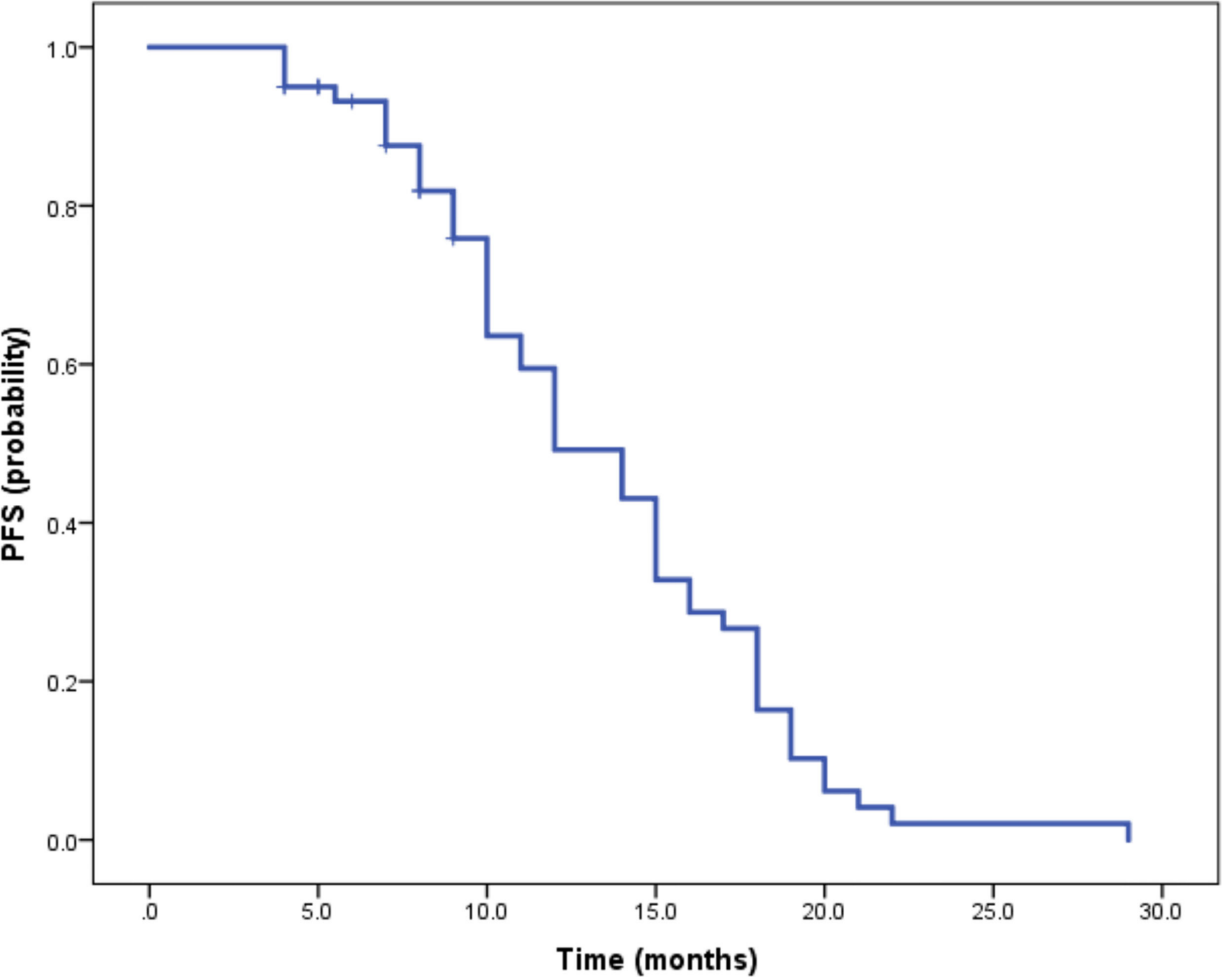
Kaplan-Meier method-based estimate of PFS by mRECIST.

**Figure 4:**
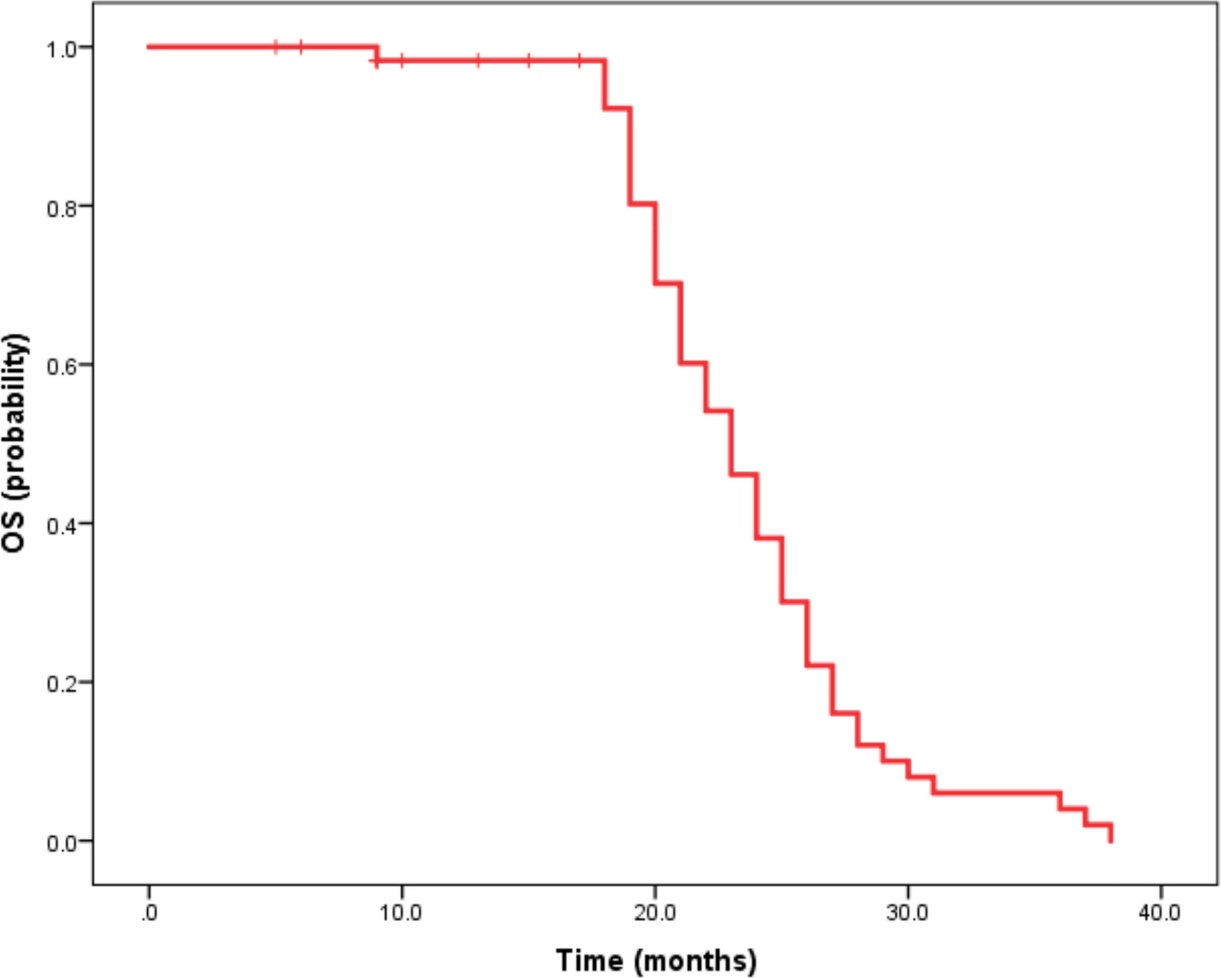
Kaplan-Meier method-based estimate of OS by mRECIST. Imaging data of a 63-year-old male patient.

**Figure 5A1–D2:**
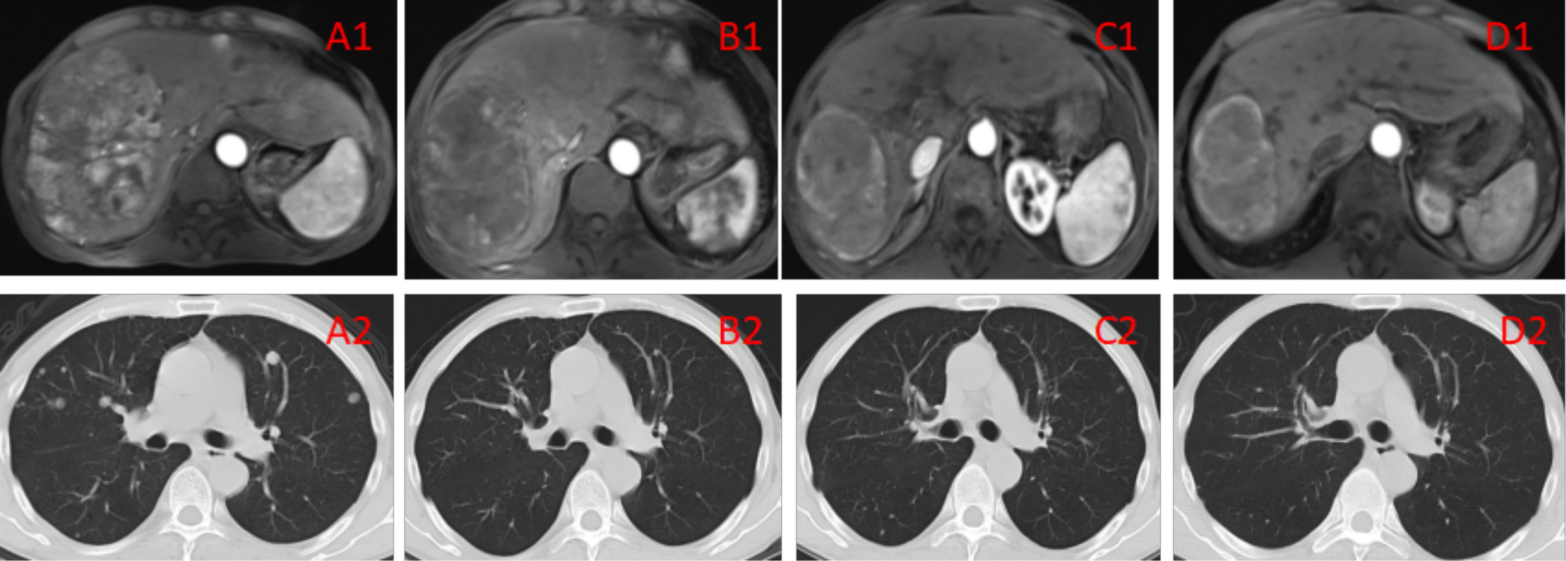
**(A1, A2)** Imaging manifestations of the patient before the treatment, showing a massive tumor accompanied by multiple small lesions in the right lobe of the liver, as well as multiple metastatic lesions in bilateral lungs. BCLC liver cancer stage: IIIB. **(B1, B2)** Imaging manifestations of the patient after 2 TACE sessions and 3 cycles of immunotherapy, showing the tumor in the right lobe of the liver with evident necrosis compared with the pretreatment condition as well as reduced number and sizes of multiple metastatic lesions in bilateral lungs. The efficacy evaluation showed PR. **(C1, C2)** Imaging manifestations of the patient after 4 TACE sessions and 6 cycles of immunotherapy, showing that the lesion in the right lobe of the liver was generally necrotic, as well as overtly reduced number of lesions in bilateral lungs. The efficacy evaluation showed PR. **(D1, D2)** Imaging manifestations of the patient after 12 cycles of immunotherapy, showing that the lesion in the liver was generally necrotic, with no lung lesions. The efficacy evaluation showed CR.

Median duration of follow-up was 12.5 months (95%CI 9.1 to 14.8 months). Tumor assessments were based on the mRECIST criteria. Objective Response Rate (ORR) was 46.7% (28/60), with complete response (CR) and partial response (PR) observed in 4 and 24 patients, respectively. Twenty-three patients were rated as stable disease (SD), and nine had progressive disease (PD). Reductions of tumor size are shown in **Figure 1**. Median duration of response (DOR) for confirmed responders was 10.0 months (95%CI 9.0-11.0 months, **Figure 2**). Median progression-free survival (PFS) was 13.3 months (95%CI 11.9-14.7 months, **Figure 3**). Median overall survival (OS) was 23.6 months (95%CI 22.2-25.0 months, **Figure 4**). A patient was evaluated as CR after treating with TACE combined with lenvatinib plus sintilimab as shown in **Figures 5A1–5D2**.

### Safety

Totally 44 patients (84.6%) showed adverse events (AEs) of any grade (**Table 2**). The most common treatment-related AEs were fatigue (30.8%), hypertension (25%), diarrhea (19.2%), decreased appetite (23%) and Palmar-plantar erythrodysesthesia (21.1%).

**Table 2:** Treatment-related adverse effects.

AEs	Any Grade NO. (%)	Grade 1 NO. (%)	Grade 2 NO. (%)	Grade 3 NO. (%)	Grade 4 NO. (%)	Grade 5 NO. (%)
Fatigue	16 (30.8)	5	6	5	0	0
Hypertension	13 (25.0)	7	4	2	1	0
Diarrhea	10 (19.2)	5	3	2	0	0
Decreased appetite	12 (23.0)	4	5	3	0	0
Weight decreased	5 (9.6)	3	1	1	0	0
Palmar-plantar erythrodysesthesia syndrome	11 (21.1)	5	3	2	1	0
Proteinuria	2 (3.8)	0	0	1	1	0
Nausea	2 (3.8)	1	1	0	0	0
Thrombocytopenia	1 (1.9)	0	0	1	0	0
Abdominal pain	5 (9.6)	2	2	1	0	0
Hypothyroidism	5 (9.6)	2	2	1	0	0
Rash	4 (7.7)	1	3	0	0	0
Abnormal liver function	1 (1.9)	0	0	0	0	1
Alimentary tract hemorrhage	1 (1.9)	0	0	0	0	1

During the study, 48% of patients showed grade 3 AEs [n=25], with hypertension as the most common grade 3 event (24%); 5.7% of patients had grade 4 AEs [n=3]. Totally 3.8% of patients developed grade 5 AEs, including abnormal liver function (n=1) and alimentary tract hemorrhage (n=1). A total of 11.5% (n=6) of patients discontinued any treatment component because of adverse events; one patient died from treatment related alimentary tract hemorrhage on day 134.

## Discussion

Theraprutic options for unresectable HCC have been developed rapidly in recent years. Sorafenib was the only available systemic therapeutic over a decade ago, and lenvatinib has also become a first−line systemic therapeutic agent for advanced HCC after the REFLECT trial (18). Although immune checkpoint inhibitors alone do not achieve a very significant effect, the combination of immunotherapy and systemic therapy could be very satisfactory. In a phase Ib study, lenvatinib plus pembrolizumab showed promising results in patients with unresectable HCC (19). In this study the ORR reached 46.0% and 36.0% by the mRECIST and RECIST 1.1 criteria, respectively. Median DORs were 8.6 months by mRECIST and 12.6 months by RECIST v1.1. Median overall survival was 22 months. In the IMbrave150 trial (20), atezolizumab plus bevacizumab showed significantly better OS and PFS compared with sorafenib in patients with unresectable HCC. In this study, OS rates at 12 months were 67.2% and 54.6% with atezolizumab plus bevacizumab and sorafenib alone, respectively; median PFS times were 6.8 months and 4.3 months, respectively. Although the exact mechanism of this combination therapy is uncertain, it is possible that VEGF may play an important role in cancer immune evasion. VEGF can enhance immune-suppressive effects in the tumor microenvironment though 3 mechanisms (21), i.e., inhibition of DC maturation to reduce T-cell activation, reduction of T-cell tumor infiltration and increase of inhibitory cells such as myeloid derived suppressor cells (MDSCs) and regulatory T cells (Tregs).

TACE is an effective treatment option for intermediate stage (multinodular, preserved liver function and ECOG PS=0) HCC (22–24). However, repeated TACE may lead to liver function impairment and even TACE resistance (25, 26), and TACE alone is unsatisfactory for patients in advanced stage (portal invasion or extrahepatic spread). Therefore, many studies have adopted TACE combined with systemic therapy for the treatment of unresectable HCC (27). TACE combined with sorafenib and lenvatinib, respectively, are commonly used in unresectable HCC (6, 28–30). The possible mechanism is that TACE induces angiogenesis and enhances the serum concentrations of VEGF because of local hypoxia, suggesting that VEGF may exert its greatest antiangiogenic effects before or after TACE (31). More importantly, recent studies found that pre-treatment with molecular targeted agents before TACE can normalize tumor vessels and upregulate VEGF, which may lead to a homogeneous distribution of lipiodol mixed anticancer drugs in the tumors (6). In this study, in order to obtain the best results, all patients received lenvatinib 2 weeks before TACE.

In present study, the ORR was 46.7% (28/60); CR and PR were observed in 4 and 24 patients, respectively. The DOR was 10.0 months (95%CI 9.0-11.0 months). PFS and OS were 13.3 months (95%CI 11.9-14.7 months and 23.6 months (95%CI 22.2-25.0 months), respectively. The median DOR, PFS and OS were longer than reported in previous trials combining PD-1 inhibitors and lenvatinib such as the IMbrave150 study (20) and other clinical trials (19). Therefore, this study showed that TACE combined with lenvatinib plus sintilimab is very effective in unresectable HCC.

This treatment was also safe as shown above. The most common treatment-related AEs were fatigue, hypertension, diarrhea, decreased appetite and Palmar-plantar erythrodysesthesia. In this study, 48%, 5.7% and 3.8% of patients had grade 3, 4 and 5 AEs, respectively. Totally 11.5% of patients discontinued any treatment component because of adverse events, and one individual died because of treatment related alimentary tract hemorrhage. The above results were comparable to those reported in previous studies examining combined treatments for unresectable HCC (19, 20, 32, 33), suggesting satisfactory safety and tolerability for this combination.

There were several limitations in this study. Firstly, this was a retrospective trial with a limited sample size, which may lead to potential bias. Besides, most patients in the current study had HBV infection, and prospective multicenter studies with other etiologies are required to validate these findings. Lastly, this was a one-arm study, without a control group. Randomized controlled trials of TACE combined with lenvatinib plus sintilimab versus lenvatinib plus sintilimab should be performed to confirm the efficacy and safety of this regimen.

In conclusion, the objective response rate, duration of response, progression-free survival and overall survival in this study were satisfactory, and adverse events were manageable. Therefore, TACE combined with lenvatinib plus sintilimab is very effective in unresectable HCC.

## Data Availability Statement

The original contributions presented in the study are included in the article/supplementary material. Further inquiries can be directed to the corresponding authors.

## Author Contributions

All authors listed have made a substantial, direct, and intellectual contribution to the work, and approved it for publication.

## Funding

This study was sponsored by Medicine and Health Discipline Platform Project of Zhejiang Province (2018RC019), Medicine and Health Science Project of Zhejiang Province (2020KY483), Project of Hubei Chen Xiaoping Science and Technology Development Foundation (2020CXPJJH12000008-13) and Science Project of Beijing Medicine and Health Foundation (2020JWJKJJHKYJJ-LC19004).

## Conflict of Interest

The authors declare that the research was conducted in the absence of any commercial or financial relationships that could be construed as a potential conflict of interest.

## Publisher’s Note

All claims expressed in this article are solely those of the authors and do not necessarily represent those of their affiliated organizations, or those of the publisher, the editors and the reviewers. Any product that may be evaluated in this article, or claim that may be made by its manufacturer, is not guaranteed or endorsed by the publisher.
